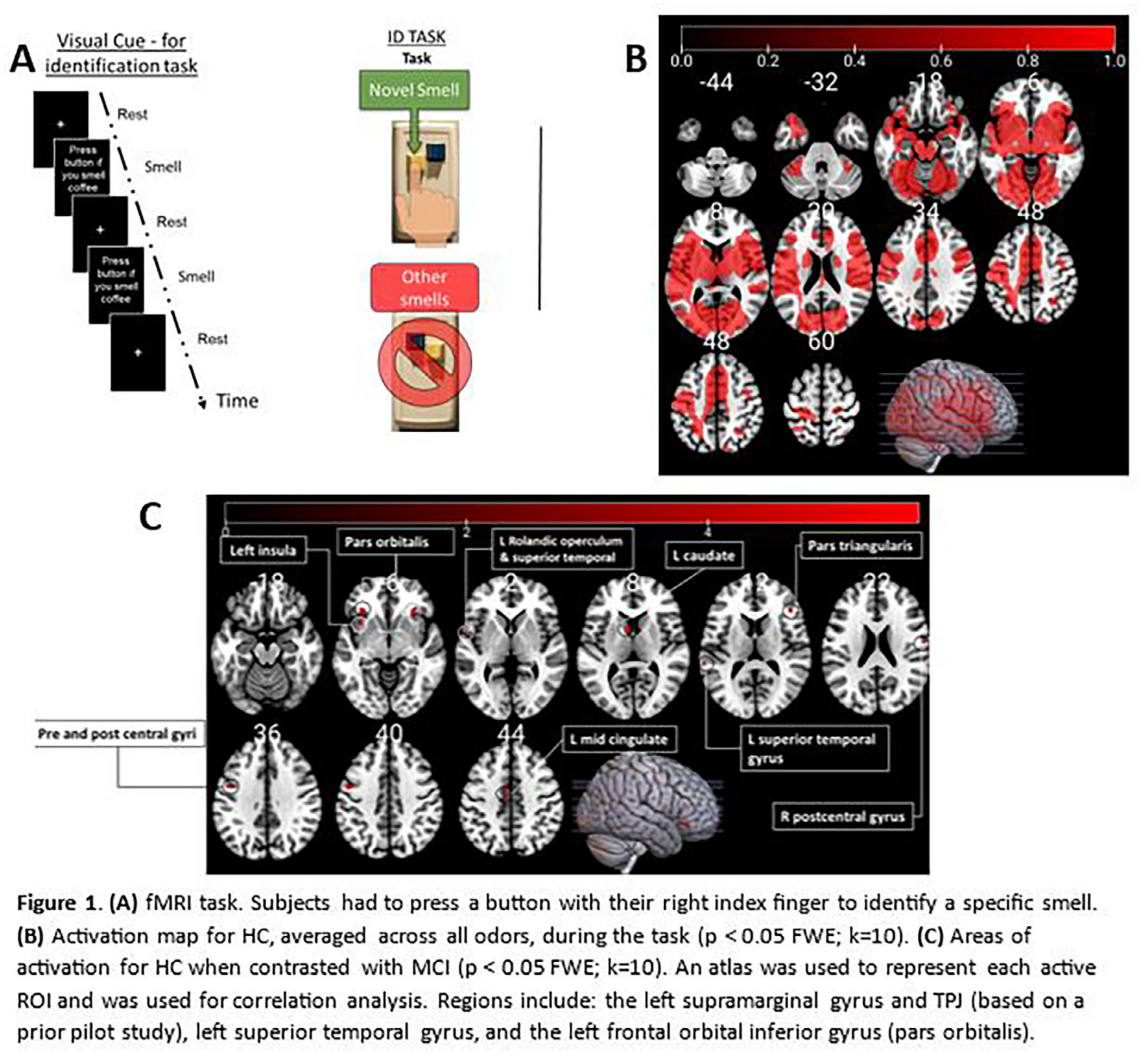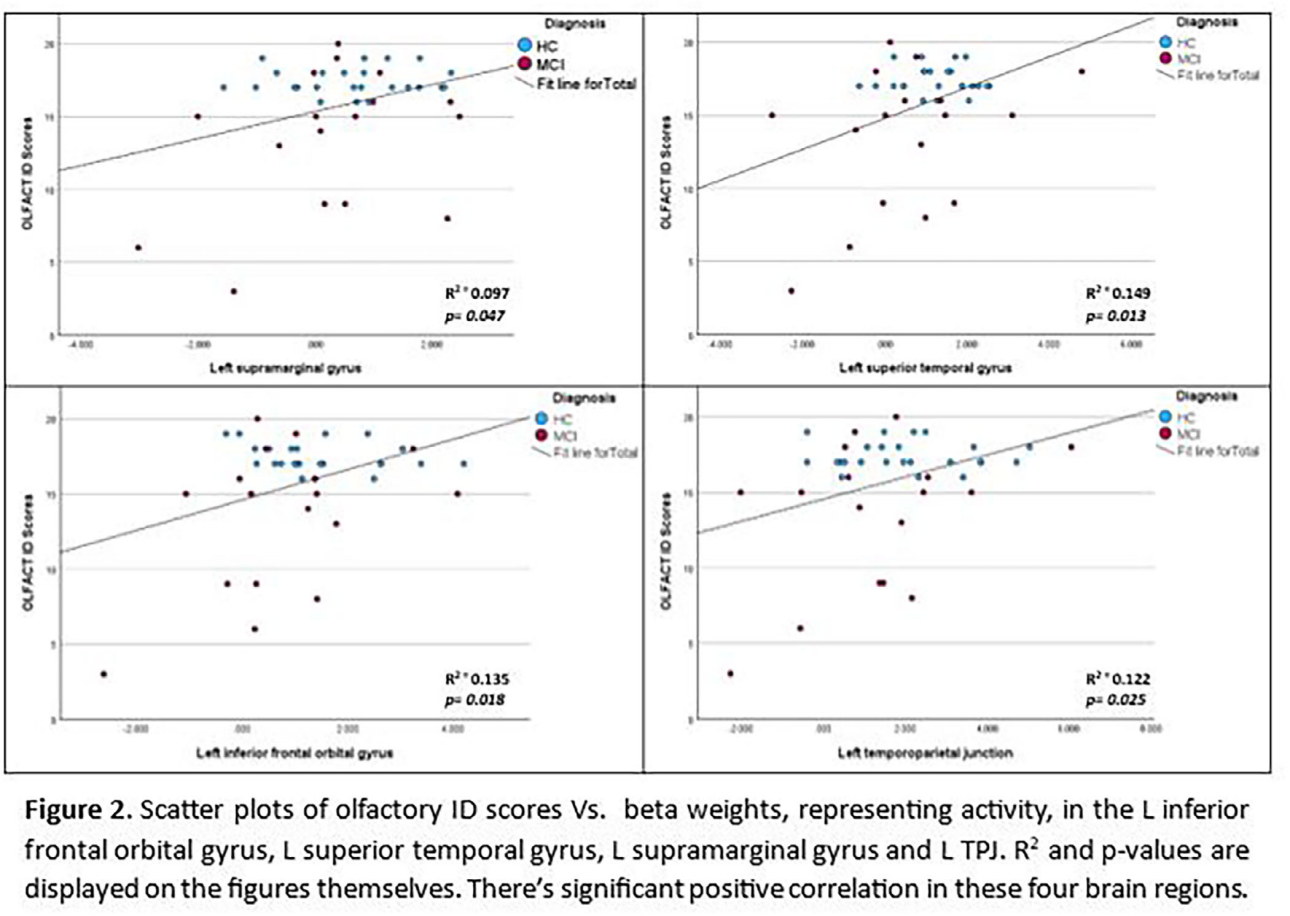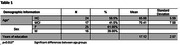# The neural substrate linking olfactory deficits to cognitive decline in MCI

**DOI:** 10.1002/alz.093347

**Published:** 2025-01-09

**Authors:** Prasanna Karunanayaka, Rommy Elyan, Biyar Ahmed, Sangam Kanekar, Senal Peiris, Ran Pang, Paul Eslinger, Qing Yang

**Affiliations:** ^1^ Pennsylvania State University College of Medicine, Hershey, PA USA; ^2^ Pennsylvania State University College of medicine, Hershey, PA USA

## Abstract

**Background:**

A combination of impairments in odor threshold and discrimination, which are both predominantly sensory level functions, and olfactory cognition contribute to age‐related odor‐identification deficits. However, in Alzheimer’s disease (AD), odor identification is more impaired compared to sensory‐level olfactory functions. We, therefore, hypothesized that match and mismatch olfactory fMRI tasks could be used to isolate areas of odor‐identification and that these areas would include primary as well as secondary olfactory structures. We combined functional magnetic resonance imaging (fMRI) with an olfactory detection task to investigate the neural substrates underlying odor identification.

**Method:**

Twenty‐five healthy controls and eighteen MCI subjects (Table 1) took part in an olfactory fMRI odor detection task. The task required subjects to press a button with their right index finger to identify an odorant (i.e., oddball) from a list of distractors. An out of magnet olfactory identification and threshold test was also conducted. This multiple‐choice test was used to measure a participants’ ability to identify twenty different odors.

**Result:**

A contrast of HC>MCI, for all odor conditions, yielded activation in brain regions shown in Figure 1C. These regions were used in a subsequent correlation analysis. The left supramarginal gyrus was included based on a study where the TPJ area (which includes parts of the supramarginal and superior temporal gyrus) was highly active in the left hemisphere. A correlation analyses using both HC and MCI showed significant positive correlations between activity in the L supramarginal, L superior temporal, L TPJ and L inferior frontal orbital areas, and out‐of‐magnet olfactory identification scores (Figure 2).

**Conclusion:**

Our novel fMRI task elicited robust activation in higher‐order olfactory structures. Since Lower activation in these regions correlated with lower ID scores across the entire cohort. HC shows greater activity in higher order brain regions, suggesting an impaired top‐down identification mechanism in MCI. There seems to be a separation between HC and MCI in Figure 2. This will be further investigated using a larger sample size.